# The -2518A/G Polymorphism in the *MCP-1* Gene and Tuberculosis Risk: A Meta-Analysis

**DOI:** 10.1371/journal.pone.0038918

**Published:** 2012-07-30

**Authors:** Yonggang Zhang, Jie Zhang, Lingjun Zeng, Honglang Huang, Min Yang, Xiaowei Fu, Can Tian, Zhangpeng Xiang, Jin Huang, Hong Fan

**Affiliations:** 1 Department of Respiratory Medicine, West China Hospital of Sichuan University, Chengdu, Sichuan, China; 2 Key Laboratory of Laboratory Medicine, Ministry of Education, Zhejiang Provincial Key Laboratory of Medical Genetics, Wenzhou Medical College, Wenzhou, Zhejiang, China; 3 Department of Respiratory Medicine, The First Hospital of Ziyang City, Ziyang, Sichuan, China; 4 West China Medical School, Sichuan University, Chengdu, Sichuan, China; 5 Department of Intensive Care Unit, The Second Hospital of Anhui Medical University, Hefei, Anhui, China; Louisiana State University, United States of America

## Abstract

**Background:**

The -2518A/G polymorphism in the *monocyte chemoattractant protein-1* (*MCP-1*) gene has been implicated in the susceptibility to tuberculosis (TB), but the results are not conclusive. The aim of this study is to investigate the association between the -2518A/G polymorphism in the *MCP-1* gene and the risk of tuberculosis by meta-analysis.

**Methods:**

We searched Pubmed, Embase, CNKI and Wanfang databases, covering all studies until April 29^th^, 2011. Statistical analyses were performed using the Revman4.2 and STATA10.0 software.

**Results:**

A total of 5341 cases and 6075 controls in 13 case-control studies were included in the meta-analysis. The results indicated that the GG homozygote carriers had a 67% increased risk of TB compared with the A allele carriers (GG vs. GA+AA: OR = 1.67, 95%CI = 1.25–2.23, P = 0.0006). In the subgroup analysis by ethnicity, significant elevated risks were found in Asians and Latinos, but not in Africans (GG vs. GA+AA: OR = 1.79, 95%CI = 1.19–2.70 and P = 0.005 for Asians; OR = 2.15, 95%CI = 1.32–3.51 and P = 0.002 for Latinos; OR = 1.28, 95%CI = 0.45–3.64 and P = 0.65 for Africans).

**Conclusion:**

This meta-analysis suggested that the -2518A/G polymorphism of *MCP-1* gene would be a risk factor for TB in Asians and Latinos, while not in Africans.

## Introduction

Tuberculosis (TB) is still one of the main causes of death due to infectious diseases [1], [2]. WHO estimated that there were almost 9.4 million new and relapse TB cases worldwide in 2009 [3]. It is reported that one third of the population had ever been infected by *Mycobacterium Tuberculosis*(MTB), but only 10% of the infected population might develop to TB with clinical symptoms [4]. Both the MTB and the host immune response may play important roles in the development of TB [5]. Numerous studies have been performed on the association of genetic variants with TB susceptibility [4], [6], [7], [8], [9] and among them, the *monocyte chemoattractant protein-1* (*MCP-1*) gene has been highlighted [9], [10], [11], [12], [13], [14], [15], [16], [17], [18], [19], [20].

**Figure 1:**
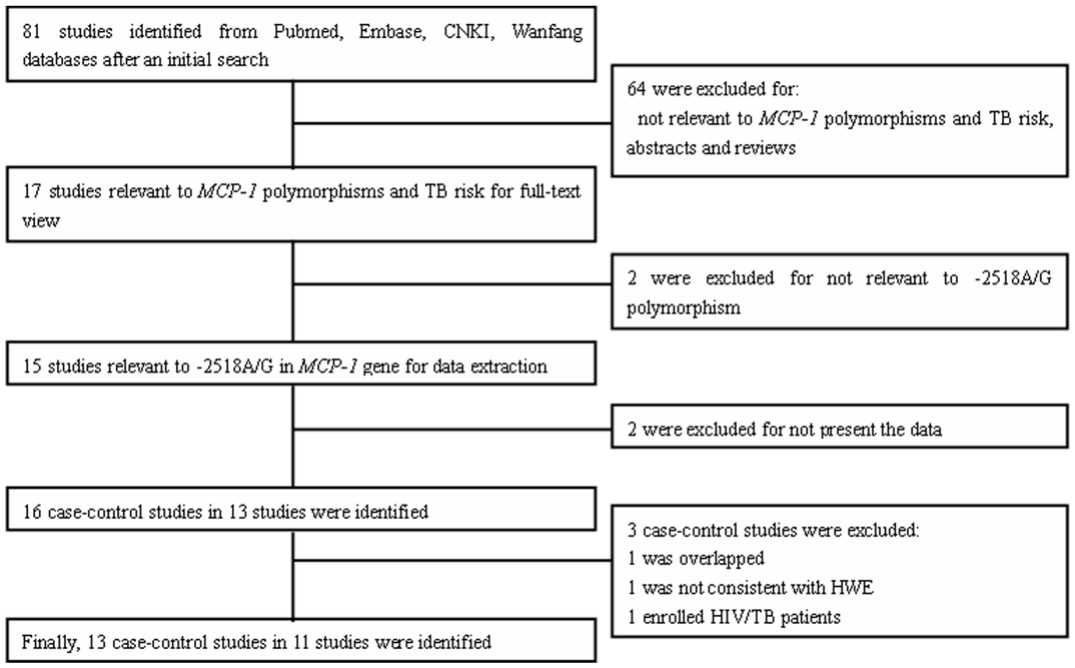
Flow diagram of included/excluded studies.

**Table 1:** Characteristics of the case-control studies included in the meta-analysis.

Author	Year	Country	Ethnicity	Case/control	The diagnosis of TB patients	Controls	HIV status
Alagarasu, K [10]	2009	India	Asian	153/203	Clinical, radiographic and/or bacteriological observations PTB	Healthy controls	Negative
Ben-Selma, W [11]	2011	Tunisia	African	223/150	Clinical symptoms, radiographical findings, AFB, culture- and histological- confirmed PTB	Healthy controls	Negative
Chu, S F [13]	2007	China	Asian	403/461	Microbiological and histological confirmed MTB infection TB*	Blood donors	Negative
Feng, W X [9]	2011	China	Asian	301/338	Diagnosis methods not mentioned; PTB(105/301) and EPTB(196/301)	Surgical children	Negative
Flores-Villanueva, P O (M) [14]	2005	Korea	Asian	129/162	Medical, biochemical and radiological assessment, AFB and culture confirmed PTB	Healthy controls	Negative
Flores-Villanueva, P O (K) [14]	2005	Mexico	Latino	435/510	Clinical symptoms, Chest radiographic findings, AFB and culture confirmed PTB	Healthy controls	Negative
Ganachari, M (M) [15]	2010	Mexico	Latino	193/243	Clinical symptoms, Chest radiographic findings, AFB confirmed PTB	Healthy controls	Negative
Ganachari, M (P) [15]	2010	Peru	Latino	701/796	Clinical symptoms, Chest radiographic findings, AFB confirmed PTB	Healthy controls	Negative
Moller, M [16]	2009	South Africa	South African Colored	431/481	Bacteriological confirmed TB*	Healthy controls	Negative
Thye, T [17]	2009	Ghana West Africa	African	1964/2312	Smear-/culture-positive PTB	Case contacts and community members	Negative
Xu, Z E [18]	2009	China	Asian	100/100	AFB, X-ray, clinical symptoms, PPD, anti-TB treatment TB*	diarrhea and health examination children	Negative
Yang, B F [19]	2009	China	Asian	167/167	AFB, X-ray, clinical symptoms, PPD, anti-TB treatment PTB	patients of Outpatient Surgery and gynecology	Negative
Zhang, S Y [20]	2009	China	Asian	141/152	AFB, X-ray, clinical symptoms, PPD, anti-TB treatment PTB	Health examination population	Negative

PTB, pulmonary tuberculosis; EPTB, Extra pulmonary tuberculosis. *, The percent of PTB and EPTB was not mentioned in the study.

**Table 2:** Distributions of *MCP-1* genotype and allele among TB patients and controls.

Author	Case			Control			Case		Control		HWE
	AA	GA	GG	AA	GA	GG	A	G	A	G	P value
Alagarasu, K [10]	78	54	21	93	81	29	210	96	267	139	0.105
Ben-Selma, W [11]	110	87	26	93	49	8	307	139	235	65	0.645
Chu, S F [13]	93	200	110	115	233	113	386	420	463	459	0.816
Feng, W X [9]	38	157	106	51	170	117	233	369	272	404	0.400
Flores-Villanueva, P O (K)* [14]	20	63	46	66	74	22	103	155	206	118	0.862
Flores-Villanueva, P O (M)* [14]	38	168	229	124	249	137	244	626	497	523	0.605
Ganachari, M (M)**[15]	23	77	93	46	127	70	123	263	219	267	0.386
Ganachari, M (P)**[15]	74	273	354	98	371	327	421	981	567	1025	0.646
Moller, M [16]	263	142	26	270	173	38	668	194	713	249	0.170
Thye, T [17]	1355	546	63	1472	748	92	3256	672	3692	932	0.803
Xu, Z E [18]	15	49	36	41	45	14	79	121	127	73	0.770
Yang, B F [19]	21	62	84	42	83	42	104	230	167	167	0.938
Zhang, S Y [20]	16	76	49	40	77	35	108	174	157	147	0.860

* , K, Korea, M, Mexico; **, M, Mexico, P, Peru.

The human *MCP-1* gene is located on chromosome 17 (17q11.2). It encodes a protein of 76 amino acids and is 13 kd in size [21]. The MCP-1 is a member of the C–C chemokine family. It is a potent chemotactic factor for monocytes [11], [13], [14], which plays critical roles in the recruitment of macrophages and T lymphocytes for controlling the dissemination of MTB [18], [19]. High levels of MCP-1 were also detected in bronchoalveolar lavage fluid of TB patients [18], [19]. There were many polymorphisms in the region of the *MCP-1* gene, and among them one important polymorphism named -2518A/G (rs1024611) was widely studied [11]. This polymorphism is located on the promoter region of the *MCP-1* gene and may modulate the expression level of MCP-1. Compared with the -2518A allele, the *MCP-1* -2518G allele was associated with increased production of both transcription and protein translation [11], [12], [18]. Several studies have reported the association between the -2518A/G polymorphism with TB risk [9], [10], [11], [12], [13], [14], [15], [16], [17], [18], [19], [20], but the results were inconclusive. Some studies reported that the polymorphism was associated with increased risk of TB in Mexicans, Koreans, Chinese and Peruvians [14], [15], [18], while the others reported different results, such as in South African Coloreds, Indians, and Ghanaians [10], [16], [17]. Meta-analysis is a useful method for investigating the risk factors of genetic diseases, because it uses a quantitative approach to combine the results from different studies with the same topic, and can provide more reliable conclusions [22], [23]. Previously, Thye [17] performed a case-control study and meta-analysis to assess the association between the risk of TB and the -2518A/G polymorphism, but no association was found. However, only five case-control studies were included, and they might be too underpowered to identify reliable conclusions. In addition, the limitation of included studies might also restrict their subgroup analyses of the original ethnic differences. More recent studies concerning the association between the polymorphism and TB risk in different populations have not been included [9], [11], [15], [18], [19], [20]. Furthermore, several important factors which may bias the results of genetic association studies were not clearly addressed, such as, Hardy-Weinberg equilibrium (HWE). The stability of meta-analysis was not discussed and different genotype comparative results were also not mentioned. Thus, we carried out a meta-analysis that included the most updated data to investigate the association between the -2518A/G polymorphism in the *MCP-1* gene and the risk of TB, and the ethnic differences. This is, to our knowledge, the most comprehensive meta-analysis regarding the -2518A/G polymorphism and TB risk.

**Figure 2:**
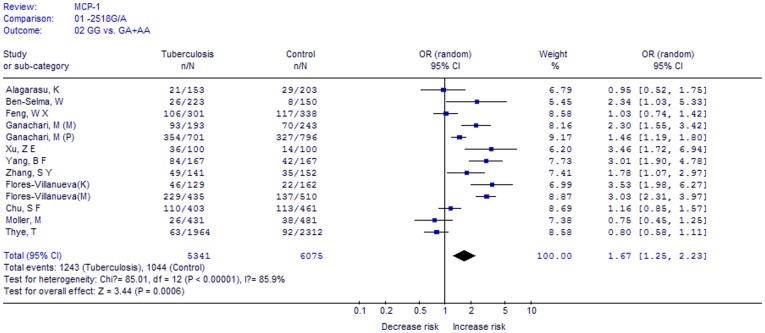
Meta-analysis with a random-effects model for the association between TB risk and the *MCP-1* -2518A/G polymorphism (GG vs. AA+GA).

## Materials and Methods

### 1. Study identification and selection

A systematic literature search in Pubmed database, Embase database, Wanfang database (www.wanfangdata.com.cn) and CNKI database (China National Knowledge Infrastructure, www.cnki.net) were carried out to identify studies involving the association between TB risk and *MCP-1* polymorphisms on April 29^th^, 2011. The search terms were as follows: ‘TB or tuberculosis’ in combination with ‘polymorphism or variant or mutation’ and in combination with ‘Monocyte chemoattractant protein-1 or MCP-1 or CCL2 or Chemokine (C–C motif) ligand 2′. The languages were limited to English and Chinese. Inclusion criteria were defined as follows: (a) studies evaluated the association between *MCP-1* -2518A/G and TB risk; (b) the design had to be a case-control study based on unrelated individuals; (c) sufficient data (genotype distributions for cases and controls) was available to estimate an odds ratio (OR) with its 95%CI; (d) genotype distributions in control group should be consistent with HWE. Studies were excluded if one of the following existed: (a) the design based on family or sibling pairs, (b) the genotype frequencies or number not reported, (c) reviews and abstracts, (d) studies with HIV/TB patients. If more than one study by the same authors using the same case series was published, either the studies with the largest sample size or the ones that were published most recently were included. The supporting PRISMA checklist is available as supporting information; see Checklist S1.

**Figure 3:**
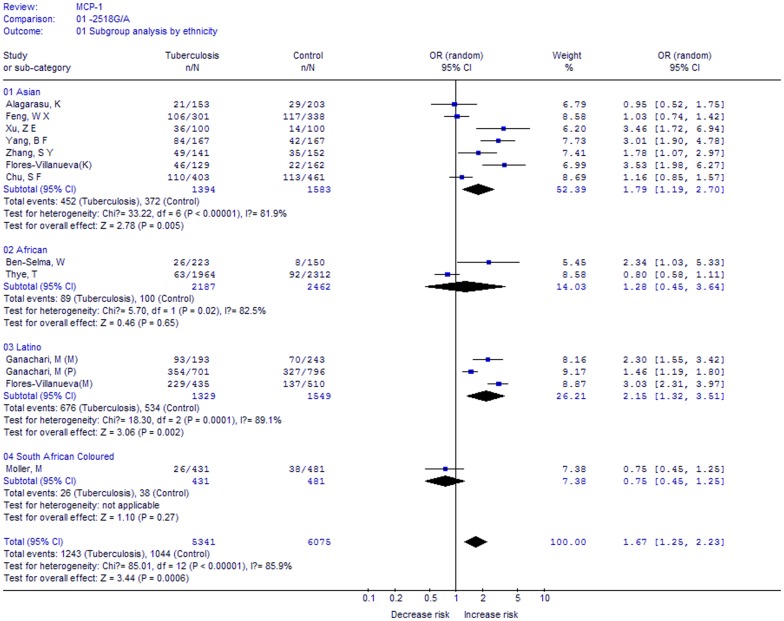
Meta-analysis with a random-effects model for the association between TB risk and the *MCP-1* -2518A/G polymorphism (GG vs. AA+GA): subgroup analysis by ethnicity.

### 2. Data extraction

Two reviewers collected the data and reached a consensus on all items. The following items were extracted from each study if available: first author, year of publication, country of origin, ethnicity, sample size, TB definition, genotyping method, and genotype number in cases and controls.

**Table 3:** Summary of results from different comparative genetic models.

-2518A/G	N^a^	Cases/Controls	GG vs. GA+AA		GG+GA vs. AA		G vs. A	
			OR(95%CI)	P^b^	OR(95%CI)	P^b^	OR(95%CI)	P^b^
Total	13	5341/6075	1.67(1.25, 2.23)	0.0006	1.60(1.17, 2.18)	0.003	1.46(1.15, 1.86)	0.002
Asian	7	1394/1583	1.79(1.19, 2.70)	0.005	1.87(1.18, 2.95)	0.007	1.58(1.14, 2.18)	0.005
Afrian	2	2187/2462	1.28(0.45, 3.64)	0.65	1.12(0.53, 2.34)	0.77	1.13(0.58, 2.24)	0.72
Latino	3	1329/1549	2.15(1.32, 3.51)	0.002	1.90(0.97, 3.72)	0.06	1.76(1.16, 2.68)	0.008
South African Colored	1	431/481	0.75(0.45, 1.25)	0.27	0.82(0.63, 1.06)	0.14	0.83(0.67, 1.03)	0.09

SAC: South African Coloureds. a, number of case-control studies; b, p value for z-test.

### 3. Statistical analysis

The strength of the association between the *MCP-1* -2518A/G polymorphism and the risk of TB was measured by OR and 95% CI. The recessive genetic model (GG vs. AG+AA) was used to evaluate the pooled OR. OR was analyzed by fixed-effects model or random-effects model according to the results of heterogeneity [24]. Heterogeneity was evaluated by a *X^2^* based *Q* statistic and was considered statistically significant when *P<*0.10. When the *P* value was >0.10, the pooled OR was calculated by the fixed-effects model, otherwise, the random-effects model was used. The significance of the pooled OR was determined by the *Z*-test and was considered statistically when the *P* value was less than 0.05. To evaluate the ethnicity-specific effects, subgroup analyses by ethnic groups were performed. For this polymorphism, other genetic models (GG+GA vs. AA and G vs. A) were also used to assess the association with the risk of TB.

**Figure 4:**
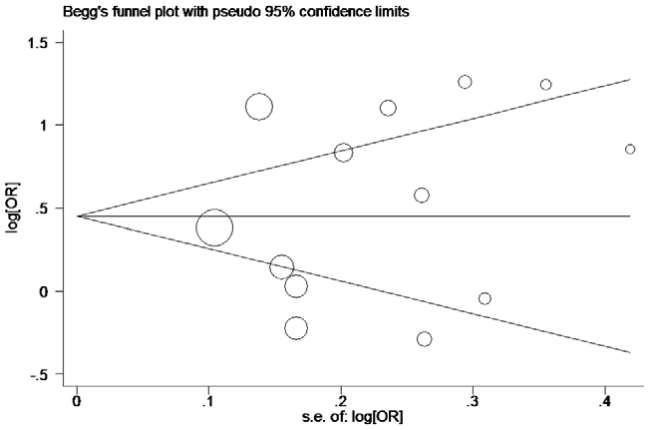
Begg's funnel plot for publication bias in selection of studies on the *MCP-1* -2518A/G polymorphism (GG vs. AA+GA).

Publication bias was analyzed by Begg's funnel plots and Egger's test [24]. Sensitivity analysis was performed by sequentially excluding individual study to assess the stability of the results [24]. HWE was tested by Pearson's *X^2^* test (P<0.05 means deviated from HWE). All statistical tests were performed using the Revman4.2 and STATA 10.0 software.

## Results

### 1. Studies selection process and characteristics

As it shows in Figure 1, a total of 81 results were identified after an initial search. After reading the titles and abstracts, 17 potential studies were included for full-text view. After reading full texts, two studies were excluded for not being relevant to TB risk and the *MCP-1* gene. Thus, 15 studies were left for data extraction. In addition, two studies were excluded for not reporting the usable data. Three studies reported two cohorts each [14], [15], [17] and each cohort was extracted as a separate case-control study. Thus, a total of 16 case-control studies in 13 studies were identified. The genotype in the control group for one case-control study was not consistent with HWE and that study was excluded. One case-control study was excluded for data overlapping or duplicated data, and one study was excluded for enrolling the HIV patients. Thus, a total of 13 case-control studies in 11 studies were identified [9], [10], [11], [13], [14], [15], [16], [17], [18], [19], [20]. Seven case-control studies were performed with Asians [9], [10], [13], [14], [18], [19], [20], two with Africans [11], [17], three with Latinos [14], [15] and one with South African Coloureds (a mixed ancestry which includes San, Khoi, Malaysians, African black and Europeans) [16]. The characteristics of each case-control study are listed in Table 1. Genotype and allele distributions for each case-control study are shown in Table 2.

### 2. Quantitative data synthesis

A total of 5341 cases and 6075 controls in 13 case-control studies were included. We analyzed the heterogeneity of GG vs. GA+AA for all 13 studies and the value of *X^2^* was 85.01 with 12 degrees of freedom and P<0.00001. Thus, we chose the random-effects model to synthesize the data. Overall, OR was 1.67 (95%CI = 1.25–2.23) and the test for overall effect Z value was 3.44 (P = 0.0006) for GG vs. GA+AA model (Figure 2). The results suggested that individuals who carry the GG homozygote may have a 67% increased risk of TB compared with the A allele carriers (AA+AG).

In the subgroup analysis by ethnicity (GG vs. GA+AA, Figure 3), significantly increased risks were found among Asians (OR, 1.79; 95%CI, 1.19–2.70; *P* = 0.005) and Latinos (OR, 2.15; 95%CI, 1.32–3.51; *P* = 0.002), but not for Africans (OR, 1.28; 95%CI, 0.45–3.64; *P* = 0.65). Thus, the results indicated that the polymorphism is associated with increased risks of TB in Asians and Latinos, but not in Africans.

A summary of results from other comparisons is listed in Table 3.

### 3. Publication bias

Publication bias was analyzed by using the Begg's funnel plots and Egger's test. The shape of the funnel plots was seemed symmetrical in the GG vs. GA+AA comparison genetic model, suggesting the absence of publication bias (Figure 4). The Egger's test was performed to provide statistical evidence of funnel plot asymmetry. The results indicated a lack of publication bias (*t* = 0.56, *P* = 0.584).

### 4. Sensitivity analysis

After sequentially excluding each case-control study, statistically similar results were obtained for GG vs. GA+AA (all P values were <0.05), suggesting the stability of this meta-analysis (data not shown).

## Discussion

It is well known that TB is a complex infectious disease that involves the infection of MTB, host immune response and gene-environment interactions. There is different individual susceptibility to TB reported. Host genetic factors, including variants of susceptible genes involved in the pathogenesis of TB, may affect these differences [4], [7], [25]. Therefore, more interest focusing on the genetic susceptibility to TB has led to a growing attention in recent years. The -2518A/G polymorphism in the *MCP-1* gene is one of the most widely studied polymorphisms among them. A growing number of studies have indicated that this polymorphism is associated with the increased risk of TB. However, the results from different published studies were inconsistent. Thus, we performed this meta-analysis to comprehensively analyze these associations. To our knowledge, this is the largest meta-analysis to date investigating the association between the *MCP-1* -2518A/G polymorphism and TB risk.

Compared with the previous case-control study and meta-analysis by Thye [17], our meta-analysis included a total of 13 case-control studies with 5341 cases and 6075 controls, which doubled the cases compared to the study mentioned above. In previous meta-analysis, the authors did not find a significant association, while we found a significant association between this polymorphism and TB risk in our study. There are several reasons that may explain the different results between our study and Thye's study. First, our meta-analysis included more case-control studies than Thye's study, thus our study is more powerful and the conclusion may be more reliable. Second, the studied populations came from more regions with different genetic backgrounds than previous meta-analysis, so our results might be more conclusive. Third, only allelic frequencies were compared in Thye's study, while the comparisons of all genetic models were performed in our study. In total, these results indicate that this polymorphism may contribute to TB pathogenesis, and help to explain individual differences in host susceptibility to TB.

After subgroup analyses according to ethnicity, we found that the variant GG homozygote carriers had a 79% increased risk of TB in Asians and 115% in Latinos, but not in Africans. It is possible that different genetic backgrounds may account for these differences. In addition, TB is a complex disease that resulted from the infection of MTB and genetic susceptibility. It is possible that these differences in genetic susceptibility might also be owed to individual status or other factors [24]. Thus, further studies are needed to assess the effect of more interactions in different ethnicities and to validate our results.

We must also mention that heterogeneity is important in meta-analysis. In our meta-analysis, significant heterogeneity was found in the overall analysis. There are several factors accounting for heterogeneity. Firstly, the genetic backgrounds for cases and controls may account for it. Different populations have different genetic backgrounds, which contribute to genetic heterogeneity. Secondly, environmental exposures in different case-control studies were not investigated, and these may also influence genetic susceptibility. Thirdly, the cases and controls included were not very homogenous in the meta-analysis: some case-control studies included both pulmonary TB (PTB) patients and extra-pulmonary TB (EPTB) patients and some included PTB only; some studies employed healthy populations as controls, while some studies employed surgical patients as controls. Thus, the results should be considered with caution, and in the future, more studies should be performed to assess these results.

Some limitations should be acknowledged when explaining the results. Firstly, only published data which were included by the selected databases were included; it is possible that some relevant published studies or unpublished studies which had null results were missed, which might bias the results, while our statistical tests may not have totally shown it. Secondly, all case-control studies were from Asians, Africans, Latinos and South African Coloureds; thus, the results may be applicable to these ethnic populations only. Thirdly, data were not stratified by other factors such as gender status and the clear diagnosis methods of TB, because sufficient information could not be extracted from the original studies. Fourth, we should pay attention to the control population, as it is unknown whether the controls had ever been infected by MTB or not, we also could not exclude the possibility that some of the controls were latent TB infection (LTBI) and they might develop active TB in future.

Despite of these limitations, this study also has some advantages. First, it updates the recent data for this polymorphism and TB risk. Second, it is the first time studying the ethnic specificity and *MCP-1* -2518A/G polymorphism interactions. Third, the methodological issues for meta-analysis, such as, heterogeneity, publication bias, and stability of results were all well investigated.

In conclusion, this meta-analysis suggested that the *MCP-1* -2518A/G polymorphism is associated with increased risk of TB, especially in Asians and Latinos. More studies with a larger group of populations should be performed to analyze these associations, especially in Africans and Caucasians.

## Supporting Information

Checklist S1 **PRISMA Checklist.**
(DOC)
